# Nanomaterials for Modified Asphalt and Their Effects on Viscosity Characteristics: A Comprehensive Review

**DOI:** 10.3390/nano14181503

**Published:** 2024-09-16

**Authors:** Hualong Huang, Yongqiang Wang, Xuan Wu, Jiandong Zhang, Xiaohan Huang

**Affiliations:** 1 Zhejiang Hongtu Traffic Construction Co., Ltd., Hangzhou 310052, China; hhualong@zjjtgc.com (H.H.); wyongqiang@zjjtgc.com (Y.W.); wxuan@zjjtgc.com (X.W.); 2 College of Civil Engineering and Architecture, Zhejiang University, Hangzhou 310058, China

**Keywords:** nanomaterials, modified asphalt, viscosity

## Abstract

The application of nanomaterials as modifiers in the field of asphalt is increasingly widespread, and this paper aims to systematically review research on the impact of nanomaterials on asphalt viscosity. The results find that nanomaterials tend to increase asphalt’s viscosity, enhancing its resistance to high-temperature rutting and low-temperature cracking. Zero-dimension nanomaterials firmly adhere to the asphalt surface, augmenting non-bonding interactions through van der Waals forces and engaging in chemical reactions to form a spatial network structure. One-dimensional nanomaterials interact with non-polar asphalt molecules, forming bonds between tube walls, thereby enhancing adhesion, stability, and resistance to cyclic loading. Meanwhile, these bundled materials act as reinforcement to transmit stress, preventing or delaying crack propagation. Two-dimensional nanomaterials, such as graphene and graphene oxide, participate in chemical interactions, forming hydrogen bonds and aromatic deposits with asphalt molecules, affecting asphalt’s surface roughness and aggregate movement, which exhibit strong adsorption capacity and increase the viscosity of asphalt. Polymers reduce thermal movement and compact asphalt structures, absorbing light components and promoting the formation of a cross-linked network, thus enhancing high-temperature deformation resistance. However, challenges such as poor compatibility and dispersion, high production costs, and environmental and health concerns currently hinder the widespread application of nanomaterial-modified asphalt. Consequently, addressing these issues through comprehensive economic and ecological evaluations is crucial before large-scale practical implementation.

## 1. Introduction

Traditional asphalt pavement materials often suffer from diseases such as high-temperature ruts and low-temperature cracking when subjected to increasing traffic volume and heavy loads. Apart from being related to design and construction, these issues are significantly influenced by the inherent properties of the asphalt material. Among them, the viscosity is particularly crucial, which reflects the flow resistance generated by the interaction of asphalt molecules, representing the ability to resist deformation under external forces [1,2]. Consequently, modifying regular asphalt has become a common method to enhance road performance. In recent years, nanotechnology has garnered great attention in the transportation sector, with a crucial research focus being the modification of asphalt using nanomaterials [3,4]. The new concept of nanomaterial engineering was formally introduced by the American Materials Research Society in 1994. And a report on the application of nano-modification technology in asphalt binders was released by the Swedish Joint Laboratory for Materials Testing and Research in August 2006. Furthermore, the European Asphalt Technology Association listed “Nanotechnology in Asphalt” as one of the subjects in June 2010.

**Figure 1:**
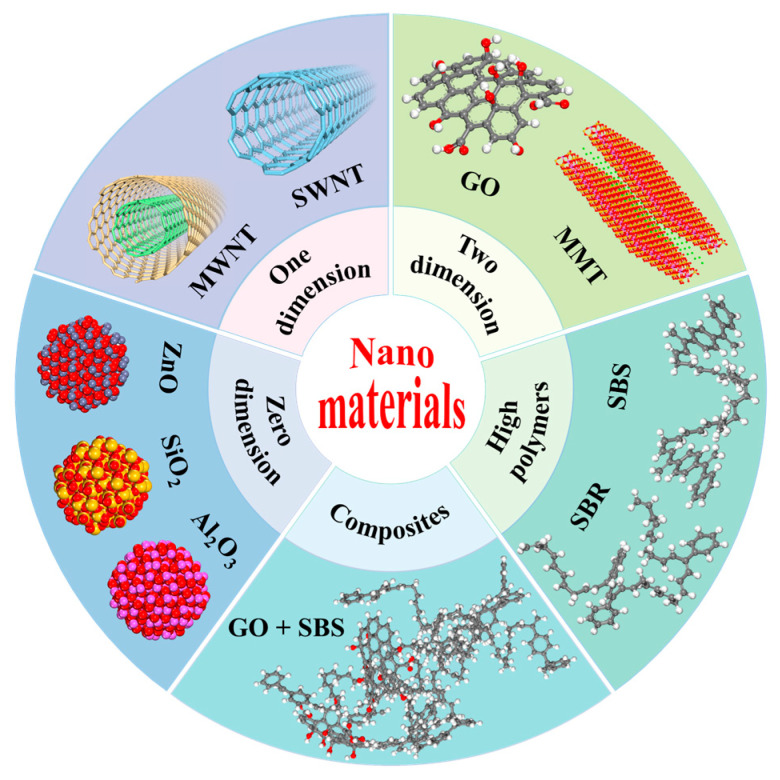
Classification of nano-modified materials.

Nanomaterials are defined as materials in which at least one dimension is within the nanoscale range (1~100 nm) or composed of such units [5]. Based on different dimensions, nanomaterials can be classified into several categories: zero-dimensional (0D) nanomaterials, such as nano-ZnO (NZ) and nano-SiO_2_ (NS) particles [6,7]; one-dimensional (1D) nanomaterials, such as carbon nanotubes (CNTs) [8]; two-dimensional (2D) nanomaterials, such as graphene oxide (GO) and nano-clay (NC) [9,10]; and high polymers, such as styrene-butadiene-styrene (SBS) and styrene-butadiene rubber (SBR) [11,12]. The classification of nanomaterials is shown in Figure 1, and properties can be seen in Table 1. Equipped with unique properties, such as volume effects, surface effects, quantum size effects, macroscopic quantum tunneling effects, and dielectric confinement effects [5], nanomaterials can effectively enhance the performance of asphalt at the microscopic scale, addressing rutting, cracking, and loosening of asphalt pavement in high and low-temperature environments. Currently, many nanomaterials have gradually achieved industrial production and are cost-effective, providing feasibility and prospects for their use as asphalt modification materials. Through databases such as PubMed and Web of Science and specific keywords including nanomaterials, asphalt viscosity, NZ, and graphene, approximately 140 articles were meticulously selected to ensure comprehensive coverage of relevant literature. The selection criteria primarily focused on papers published in reputable journals within the past decade, with a specific emphasis on topics related to asphalt rheology, nanomaterial modifiers, and their effects on viscosity-related properties.

This review stands out for its comprehensive coverage of the most commonly used nanomaterials and high polymers in asphalt modification, providing detailed insights into their viscosity characteristics and modification mechanisms. Unique to this review is its specific focus on elucidating the effects of 0D, 1D, and 2D nanomaterials, as well as high polymers, thus offering a deeper understanding of their contributions to asphalt properties, respectively. Moreover, by not only summarizing current challenges but also identifying future research hotspots, the review provides a forward-looking perspective, distinguishing itself as a comprehensive and forward-thinking contribution to the study of nanomaterial-modified asphalt, thereby enhancing its novelty and relevance in the field.

**Table 1:** Properties of nanomaterials.

Nanomaterial	Diameter (nm)	Surface Volume Ratio (m^2^/g)	Density (g/cm^3^)	Ref.
Nano SiO_2_	20~30	130~600	2.10	[13]
	11~13	200	2.40	[14]
	-	-	2.19	[15]
	30~50	200~250	-	[16]
Nano ZnO	20~30	-	-	[17]
	20	≥40	5.60	[18]
	30	~50	-	[19]
Nano TiO_2_	20	10~45	3.90	[20]
	20	50~100	3.16	[21]
	21	35~65	3.90	[22]
Nano clay	2~13	220~270	-	[23]
	<13	-	1.98	[24]
Carbon nanotube	8~10	>200	2.10	[25]
	30	200	2.10	[26]
	10~20	165	-	[27]
Graphene oxide	10~50	50~100	-	[28]
		100~300	-	[25]
		300~450	-	[29]

## 2. Zero-Dimensional Nanomaterials

### 2.1. Nano ZnO

**Figure 2:**
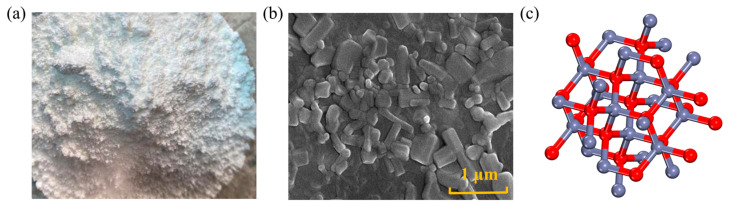
Shape and structure of NZ: (**a**) macroscopic scale; (**b**) microscale; (**c**) molecular scale. Adapted with permission from Refs. [6,36]. Copyrights 2023 and 2024 MDPI.

NZ is a white powder, as well as non-toxic, tasteless, and insoluble in water, its shape and structure are depicted in Figure 2. This material exhibits high catalytic activity and UV light absorption capacity, thus being extensively used in the development of high-performance materials such as rubber, ceramics, and coatings [6]. Adding NZ to asphalt mixtures can contribute to enhancing aging and rutting resistance. Generally, the methods for producing NZ include the sol–gel method, co-precipitation method, hydrothermal synthesis, and spray pyrolysis. Zhu [30] conducted viscosity tests, and the results indicated that within the temperature range of 110~150 °C, the surface of NZ-modified asphalt is relatively rough, as NZ is uniformly dispersed in the asphalt matrix, presenting a heterogeneous structure, which effectively increases the viscosity of asphalt. At a constant temperature, with an increase in the amount of NZ, the viscosity of modified asphalt also increases. At 135 °C, a NZ mass ratio of less than 8.0 wt.% can ensure the construction and workability of modified asphalt. Su [31] utilized molecular dynamics (MD) simulations to investigate the physical properties of NZ cluster/asphalt blends. It was found that when the NZ content was 4~5 wt.%, the physical properties of modified asphalt were superior to those of other contents. At the same time, they studied the effect of NZ on the morphology of matrix asphalt. Under atomic force microscopy (AFM), the modified asphalt exhibits a rough surface. The average roughness of the base asphalt is 0.4736, while the average roughness of NZ-modified asphalt increased by 3.6 wt.% to 0.5063. Zhang [32] used 4 wt.% as the NZ dosage and studied the effect of NZ size on asphalt viscosity through dynamic shear rheometer (DSR) tests. The results indicated that with decreasing particle size, the interaction between NZ and asphalt molecules increased, thereby enhancing the viscosity. The reason is that the smaller the NZ particles, the easier they fill the gaps between asphalt molecules, making the asphalt more compact [33,34].

The comparison of comprehensive experiments and simulations indicates that the modification mechanism of NZ on asphalt includes [34,35]: (1) The high activity and surface energy of NZ enhance its interaction with asphalt molecules, allowing NZ to firmly adsorb onto the asphalt surface and form a network structure. Additionally, as small particles, NZ disperses and fills the gaps between asphalt molecules, thereby increasing the density of asphalt and reducing molecular fluidity. (2) A portion of NZ acts as asphaltene, increasing the proportion of polar components. (3) High-speed shear generates some free radicals and unsaturated bonds in the asphalt, and the active sites and lone electrons of NZ facilitate binding with asphalt molecules, i.e., undergoing chemical reactions.

### 2.2. Nano SiO_2_

**Figure 3:**
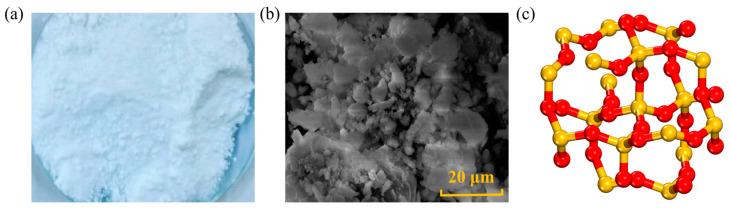
Shape and structure of NS: (**a**) macroscopic scale; (**b**) microscale; (**c**) molecular scale. Adapted with permission from Refs. [41,42]. Copyrights 2024 MDPI and 2023 Elsevier.

NS is an amorphous white powder, belonging to a non-toxic, tasteless, and non-polluting inorganic non-metal, as shown in Figure 3. Due to its large surface area, good dispersibility, strong adsorption, and excellent stability, NS is widely applied in the medical and engineering fields, such as a catalyst carrier, plastic filler, etc. [37,38]. The producing methods for NS include a sol–gel method, a reverse phase microemulsion method, and flame synthesis. Yao [39] added NS at weight ratios of 4 wt.% and 6 wt.% to base asphalt and analyzed the rheological properties of the modified asphalt through rotational viscosity tests (RV). According to the results, the viscosity of NS-modified asphalt decreases on average by 4 wt.%, which would result in lower compaction temperatures or construction energy consumption. However, some studies have yielded opposite results. Bhat [40] investigated the viscosity of modified asphalt with NS at mass ratios of 0 wt.%, 0.5 wt.%, 1 wt.%, and 3 wt.% in the temperature range of 60~135 °C, and the results indicated a continuous increase in viscosity with the increase in NS content. Liu [7] evaluated the viscosity of the original asphalt and NS-modified asphalt using the phase angle *δ* as an indicator, as increasing *δ* means increasing material viscosity. At the same temperature, for nustar 76-22 asphalt, at 25 °C and 22 °C, the phase angle δ of modified asphalt with a NS content of 7 wt.% increased by 11.4 wt.% and 11.5 wt.%, respectively, compared to the base asphalt, indicating that the addition of NS increased the viscosity of the asphalt.

The modification mechanism of NS mainly involves: (1) The surface’s free hydroxyl groups and silane coupling hydroxyl groups enable NS to form hydrogen bonds with the polar components in the asphalt, thereby improving their compatibility. (2) Through specific dispersion means, NS can penetrate near the π bonds of polymer compounds, overlap with their electron clouds, and form a spatial network structure [43].

Based on these results, the effect of NS on asphalt viscosity is not universally consistent and is influenced by various parameters, which may depend on NS concentration, temperature, and specific properties of the base asphalt. Obviously, this emphasizes the necessity of further research to comprehensively understand the relationship between NS content and asphalt viscosity under different conditions.

### 2.3. Nano TiO_2_

**Figure 4:**
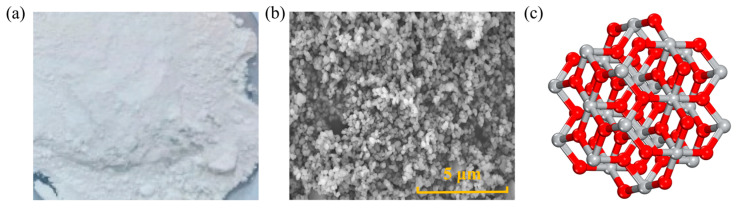
Shape and structure of NT: (**a**) macroscopic scale; (**b**) microscale; (**c**) molecular scale. Adapted with permission from Refs. [41,48]. Copyrights 2024 and 2023 MDPI.

Nano TiO_2_ (NT) is an inorganic compound, that appears as a white powder, is insoluble in water, and serves as an excellent photocatalytic material. It primarily exists in three forms in nature: rutile, anatase, and brookite. Currently, the NT used is predominantly sourced from anatase due to its abundant reserves [44], as depicted in Figure 4. Shafabakhsh [45] added NT contents of 1 wt.%, 3 wt.%, 5 wt.%, and 7 wt.% of asphalt weight and determined viscosity through RV tests at 135 °C for both conventional and NT-modified asphalt. According to the findings, the addition of NT increased the asphalt viscosity, with 5 wt.% NT being identified as the optimal ratio. Ayar’s study [44] indicated that the addition of 1 wt.%, 3 wt.%, and 5 wt.% NT in asphalt mixtures resulted in higher viscosity, thereby enhancing adhesion and improving resistance to rutting. The optimal dosage of NT for rheological and mechanical performance varies with different dosages, and the suggested ratio was 5 wt.% of asphalt weight. Enieb [46] conducted viscosity tests, demonstrating that the addition of NT to 60/70# asphalt could provide higher rational viscosity at 135 °C. In comparison to regular TiO_2_, NT possesses a larger surface area, smaller diameter, and lower opacity, favorably contributing to the enhancement of the rheological properties of asphalt [47].

Comparatively, the optimal NT dosage for enhancing rheological and mechanical properties appears to vary, with 5 wt.% of asphalt weight emerging as a suggested ratio in multiple studies. Additionally, the unique physical characteristics of NT, such as its larger surface area, smaller diameter, and lower opacity, are highlighted as favorable attributes contributing to the enhancement of asphalt’s rheological properties.

### 2.4. Nano Al_2_O_3_

**Figure 5:**
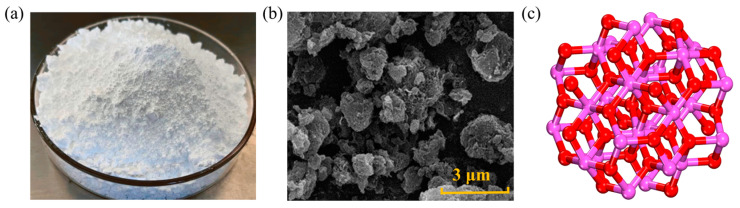
Shape and structure of NA: (**a**) macroscopic scale; (**b**) microscale; (**c**) molecular scale. Adapted with permission from Refs. [41,51]. Copyrights 2024 Elsevier and MDPI.

Nano Al_2_O_3_ (NA), with particle sizes mostly ranging from 5 to 10 nm, possesses an enormous specific surface area that can exceed 1000 m^2^/g, exhibiting high reactivity and finding extensive applications in the field of catalysis. Considering its advantages in thermal stability, chemical stability, and mechanical strength, NA has emerged as a potential asphalt modifier [49], as depicted in Figure 5. Methods for preparing NA include mechanical ball milling, laser ablation, solution reduction, and gas evaporation techniques [50]. Adnan [51] determined the zero shear viscosity (ZSV) of NA-modified asphalt at 60 °C through shear tests, revealing an increase in ZSV with an increase in NA dosage, at a concentration of 3 wt.%. However, when the dosage reached 4 wt.%, NA particles agglomerated, leading to a reduction in ZSV. Ali [52] assessed the rheological and physical properties of NA-modified asphalt, demonstrating improved resistance to fatigue cracking and high-temperature rutting deformation. When adding NA above 5 wt.% or less to asphalt, the phase angle *δ* decreases, indicating that 5 wt.% is the optimal level. Scanning electron microscopy (SEM) shows that NA is uniformly dispersed and regularly distributed in the continuous asphalt phase, and its agglomeration process occurs at 3 wt.% and 5 wt.%. 7 wt.% of the NA showed significant aggregation, which affected their dispersion in the asphalt binder. According to Bhat [53], based on Marshall stability test results, NA increased the viscosity of asphalt at 60 °C, enhancing the stability of asphalt mixtures. Furthermore, asphalt containing 2 wt.% NA exhibited the highest stability, showing a 63.12 wt.% increase compared to the control group.

Fourier transform infrared spectroscopy (FTIR) shows that NA modified asphalt does not generate new functional groups but only concentrates on carbonyl bonds (C-O), butadiene double bonds (HC=CH), and sulfoxide bonds (S-O), confirming that the structure of modified asphalt has not changed significantly compared to the matrix asphalt and indicating that NA modification of asphalt is only a physical process [52].

### 2.5. Nano CaCO_3_

**Figure 6:**
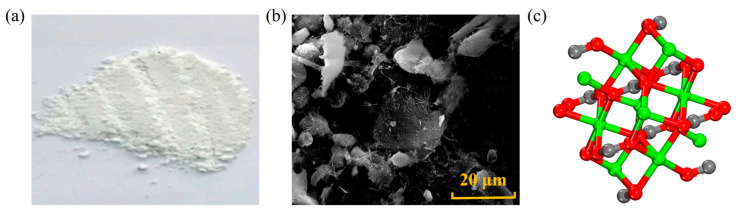
Shape and structure of NCa: (**a**) macroscopic scale; (**b**) microscale; (**c**) molecular scale. Adapted with permission from Ref. [42]. Copyright 2023 Elsevier.

Nano CaCO_3_ (NCa) is a white solid powder with an average particle size ranging from 10 to 100 nm and has been widely employed as a stable and cost-effective material for preparing modified asphalt. Its shape and structure are depicted in Figure 6. Manfro [54] utilized a Brookfield viscometer to measure the apparent viscosity of base asphalt and nano-modified asphalt with varying NCa contents. With an increase in the addition of NCa, the apparent viscosity of all samples increased. Upon adding 10 wt.% NCa, the asphalt apparent viscosity increased by approximately 49 wt.%, 28 wt.%, and 25 wt.% at temperatures of 135 °C, 150 °C, and 177 °C, respectively. Concurrently, the increase in NCa contributed to a reduction in contact angle, indicating a stronger interaction capability between the asphalt and mineral aggregates. Consequently, the incorporation of NCa resulted in increased flow resistance of the asphalt material due to the formation of an interlayer structure, thereby reducing the mobility of asphalt molecules [55].

Comparatively, this part demonstrates a substantial increase in apparent viscosity with higher NCa content. Additionally, the observed reduction in contact angle and the formation of an interlayer structure underscore the significant influence of NCa on the interaction between asphalt and aggregates, ultimately affecting the flow resistance and mobility of asphalt molecules.

### 2.6. Nano Fe_2_O_3_

**Figure 7:**
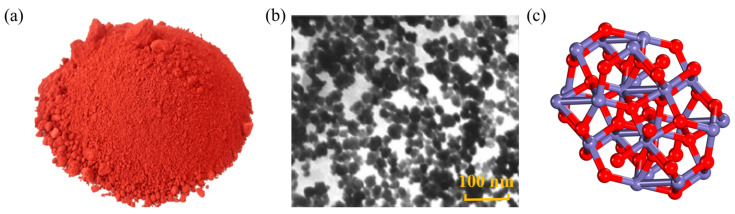
Shape and structure of NFe: (**a**) macroscopic scale; (**b**) microscale; (**c**) molecular scale. Adapted with permission from Ref. [57]. Copyright 2017 Elsevier.

Nano Fe_2_O_3_ (NFe) primarily serves as a pigment for colored road surfaces. The shape and structure of NFe are depicted in Figure 7. Shafabakhsh [56], utilizing AFM, observed that the addition of NFe resulted in a reduction in the roughness of the asphalt surface, suggesting a decrease in the stiffness properties of the modified asphalt. However, Kordi [57] indicated that as the content of NFe increased, the rut depth gradually decreased. The reason was that NFe had a large surface area, which played a filling and reinforcing role in asphalt, and improved the strength between asphalt particles. At a temperature of 40~60 °C, the modified asphalt with an NFe content of 0.9 wt.% had the strongest resistance to rutting. However, excessive addition led to negative results, as the increase in the content of nanoparticles increased the distance between asphalt molecules, thereby reducing strength and adhesion. Pirmohammad [58] indicated that as the percentage of NFe increases, the fracture resistance of asphalt initially increases, reaches its peak at 0.8 wt.%, and then decreases. According to their investigation, adding 0.9 wt.% to asphalt, the NFe exhibited the best effect on the mechanical properties of the mixture, including stiffness modulus and fatigue cracking resistance. In fact, NFe helps asphalt to produce higher adhesion, allowing aggregates to firmly bond together.

These collective findings underscore the complex relationship between NFe content and asphalt properties, indicating that while moderate additions of NFe can enhance rut resistance and mechanical properties, excessive amounts may lead to adverse effects on asphalt strength and adhesion. The part emphasizes the importance of optimizing NFe content to achieve the desired improvements in asphalt properties.

## 3. One-Dimensional Nanomaterials

One-dimensional (1D) nanomaterials utilized for asphalt modification primarily include carbon nanotube (CNT) and carbon nanofiber (CNF).

### 3.1. Carbon Nanotube

**Figure 8:**
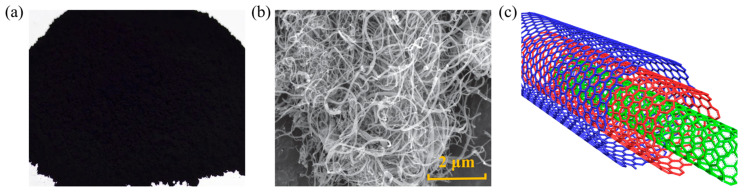
Shape and structure of CNT: (**a**) macroscopic scale; (**b**) microscale; (**c**) molecular scale. Adapted with permission from Ref. [63]. Copyright 2021 Elsevier.

CNT is a seamless cylindrical structure with a surface of sp^2^ hybridized graphite sheets, comprising two types: single-walled carbon nanotube (SWNT) and multi-walled carbon nanotube (MWNT). They exhibit outstanding properties, such as high aspect ratio, strength, thermal conductivity, and chemical resistance. With an elastic modulus of up to 1 TPa, CNT serves as an excellent asphalt modifier. The shape and structure of CNT are depicted in Figure 8, and methods for CNT preparation include arc discharge, chemical vapor deposition, and laser ablation. Zhu [59] conducted Brookfield rotational viscosity tests on CNT-modified asphalt at 135 °C, indicating that CNT increased the viscosity of the asphalt, resulting in improved elastic recovery and load-deformation properties. CNT-modified asphalt exhibits obvious fluorescence and uniform dispersion, indicating good compatibility between CNT and matrix asphalt. Fluorescent substances may undergo chemical reactions between CNTs and asphalt, generating new colloidal structures in the asphalt that enhance its network structure. Ismael [60] used CNT to enhance the high-temperature performance of asphalt and improved the resistance to rutting, as well as recommended a content of 1.5~2 wt.% CNT. Victor [61] also suggested that at a CNT content of 2 wt.%, the permanent deformation capability and fatigue resistance of asphalt mixtures could be greatly improved. Gong [62] demonstrated that CNT enhanced the resistance of asphalt binders to rutting but reduced their resistance to thermal cracking, with no chemical reactions occurring during the modification process. As the CNT content increases from 0 wt.% to 3 wt.%, the aromatic and carbonyl indices decrease, while the fat index slightly increases, indicating that CNTs not only function as nanofibers but also physically interact with asphalt binders at the molecular level. This interaction between CNTs and the asphalt matrix helps to increase the viscosity of asphalt from 0.6 Pa·s to 1.2 Pa·s.

**Figure 9:**
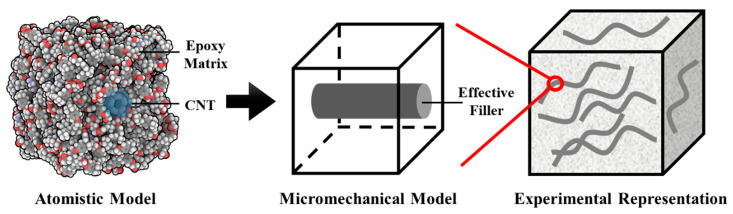
Schematic diagram of CNT distribution in asphalt. Adapted with permission from Ref. [64]. Copyright 2020 Elsevier.

The distribution of CNTs in asphalt is depicted in Figure 9. The p electrons of CNT can interact with the non-polar molecules in the asphalt to form π–σ bonds and π–π bonds between the tube walls, thereby promoting compatibility with the asphalt and enhancing adhesion and stability between the asphalt and aggregates. Simultaneously, CNT possesses high load absorption capabilities, enabling modified asphalt to exhibit excellent resistance to cyclic loading, effectively delaying the initiation and propagation of microcracks.

### 3.2. Nanofiber

**Figure 10:**
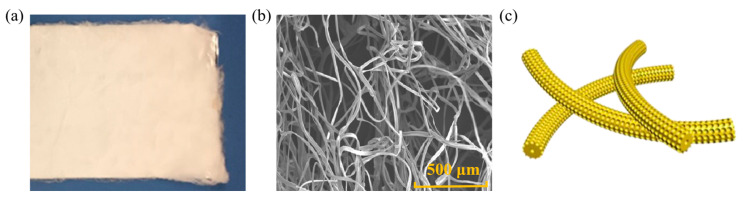
Shape and structure of nanofibers: (**a**) macroscopic scale; (**b**) microscale; (**c**) molecular scale. Adapted with permission from Refs. [66,67]. Copyrights Springer Nature and 2021 Elsevier.

Nanofibers are materials composed of continuous or discontinuous fine filaments, with a wide variety of types and extensive applications. In road materials, fibers mainly consist of carbon fibers, basalt fibers, and synthetic fibers. The shape and structure of nanofibers are illustrated in Figure 10. Khattak [65] observed the cross-sectional morphology of modified asphalt with 4 wt.% and 6.5 wt.% CNF addition using SEM and found that many fibers were pointing towards the tensile direction on the cross-section, and the fibers were interconnected to form a good network structure. With an increase in CNF content, this fiber network became denser, exhibiting stronger connectivity. Local magnification revealed the extraction behavior of CNF at the root, indicating that the CNF network can enhance the mechanical properties of asphalt and inhibit crack propagation, thereby extending its fatigue life. Ghabchi [66] analyzed the dynamic viscosity values of PG 58-28, PG 64-34, and PG 70-28 asphalts with different CNF contents at 137 °C and 167 °C. They found that the dynamic viscosity of all the asphalts increased with an increase in the CNF content. For instance, the dynamic viscosity of PG 58-28 at 137 °C (308 mPa·s) increased by 34 wt.% and 103 wt.% with the addition of 0.3 wt.% and 0.7 wt.% CNF, respectively. The impact of adding CNF to the asphalt on increasing its viscosity is more pronounced at lower temperatures.

The modification mechanism of nanofibers on asphalt performance varies according to the fiber characteristics: (1) The high porosity and absorption capability allow fibers to absorb the light components in asphalt, thereby reinforcing it. (2) Fibers with high specific surface area but do not absorb asphalt induce expansion and flocculation within the asphalt, acting as a “stabilizer”. (3) Bundle-shaped nanofibers primarily enhance and toughen the asphalt. Asphalt mixtures are heterogeneous materials with internal micro-cracks and weaker bond interfaces with aggregates, making them prone to cracking under external loads. Bundle-shaped nanofibers transmit stress within the asphalt, preventing or delaying crack propagation.

## 4. Two-Dimensional Nanomaterials

Here mainly introduce three commonly used 2D nanomaterials, namely graphene, GO, and NC (mainly montmorillonite, MMT).

### 4.1. Graphene

**Figure 11:**
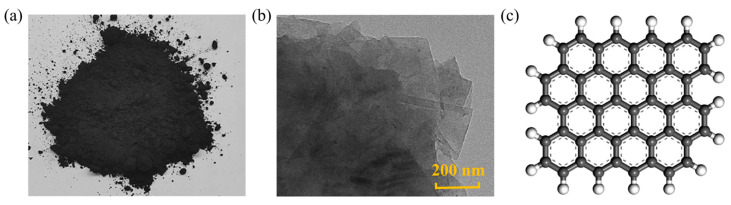
Shape and structure of graphene: (**a**) macroscopic scale; (**b**) microscale; (**c**) molecular scale. Adapted with permission from Refs. [71,72]. Copyrights 2021 and 2022 Elsevier.

Graphene is a 2D nanomaterial composed of carbon atoms connected in a honeycomb lattice structure through sp^2^ hybridization. It possesses an extremely high specific surface area, mechanical strength, flexibility, and outstanding thermal, electrical, and gas barrier properties [68,69]. Currently, graphene is considered a highly important modifier in asphalt modification as a filler, as depicted in Figure 11. Yao [70] conducted viscosity tests on control and multilayer graphene-modified asphalt at different temperatures, demonstrating that the viscosity of the modified asphalt was higher than that of the control asphalt. FTIR results indicated that the proportion of aging groups (C=O) in the modified asphalt significantly increases, leading to improved high-temperature performance and water sensitivity of the mixture. Meanwhile, the binder absorbs more radiation and energy, especially in low-temperature regions. Wang [71], using a Brookfield rotational viscometer, measured the viscosity values of original and graphene-modified asphalt. At the same temperature, the viscosity of the asphalt increased with an increase in the amount of graphene. The most significant increase in viscosity was observed when the graphene content increased from 0 wt.% to 1 wt.%. Beyond 1 wt.% graphene content, the trend of viscosity growth declined. This phenomenon can be explained by the dispersion state and lamellar structure of graphene in asphalt. When the graphene content does not exceed 1 wt.%, graphene is well dispersed in the asphalt, with nanosheets embedded and interwoven with asphalt molecular chains, effectively inhibiting the movement of asphalt molecules and leading to a rapid increase in viscosity [72]. With a continuous increase in graphene content, more agglomerates form in the asphalt, and the movement of asphalt molecules remains relatively stable, resulting in a slow increase in viscosity [73].

**Figure 12:**
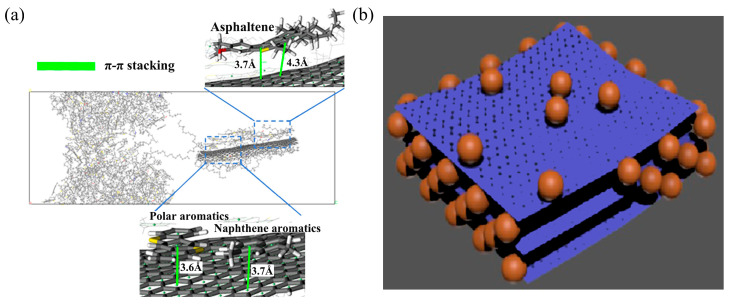
Mechanism of graphene-modified asphalt: (**a**) interface π–π interaction; (**b**) filling and barrier structure. Adapted with permission from Refs. [77,78]. Copyrights 2021 and 2018 Elsevier.

The mechanism of graphene in asphalt modification is illustrated in Figure 12, primarily including: (1) adsorption: graphene exhibits strong adsorption capability towards asphalt molecules, and the strong π–π interaction between graphene and polycyclic aromatic hydrocarbons can enhance the rheological properties of asphalt [74]; (2) filling and barrier effects: graphene, as a dispersed phase in the continuous phase of asphalt, acts as a filler. Its dense structure impedes the movement of asphalt molecules, and increases the viscosity and compactness of asphalt [75,76]; (3) gelation effect: graphene promotes the transition of the colloidal structure to a gel structure by adsorbing light components of the asphalt, and enhances the rheological properties.

### 4.2. Graphene Oxide

**Figure 13:**
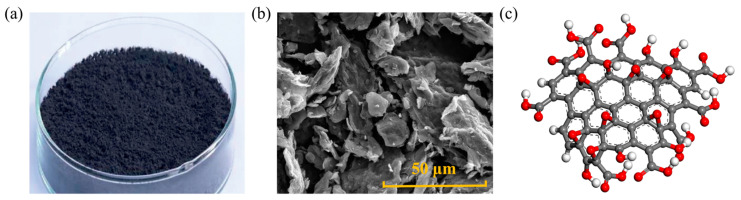
Shape and structure of GO: (**a**) macroscopic scale; (**b**) microscale; (**c**) molecular scale. Adapted with permission from Refs. [9,83]. Copyrights 2022 Hindawi and 2017 Springer.

GO possesses a unique quasi-2D layered structure with a high density of polar oxygen-containing functional groups on its surface, such as carboxyl, hydroxyl, epoxy, and ester groups, rendering it reactive and compatible with various polymeric matrices [79,80]. The shape and structure of GO are illustrated in Figure 13. Singh [28] explored the effect of GO on the dynamic viscosity of AC30 asphalt. GO was added to AC30 at different dosages of 1wt.%, 2wt.%, and 3 wt.%, showing that at all test temperatures ranging from 120 °C to 180 °C, GO increased the viscosity. This can be attributed to the predominantly aromatic structure of GO, which, through conjugated bonding, increases the polarity of AC30 and enhances molecular association. Hoang [81] assessed the viscosity of a series of asphalt samples modified with different GO concentrations (0.5 wt.%, 1 wt.%, 1.5 wt.%, 2 wt.%, and 3 wt.%), and the results demonstrated that the incorporation of GO into the asphalt matrix enhances viscosity and elasticity, increases the complex modulus, and reduces the phase angle. Among all the samples, the asphalt sample with a 2 wt.% GO concentration exhibited the highest viscosity compared to the original asphalt. Zeng [82] simulated the electron density of GO with saturates, aromatics, and resins to reflect the strength of their interactions. By comparing the binding energy, it was indicated that the interaction between GO and resin was the strongest, followed by GO and aromatics, while the interaction between GO and saturates was the weakest.

**Figure 14:**
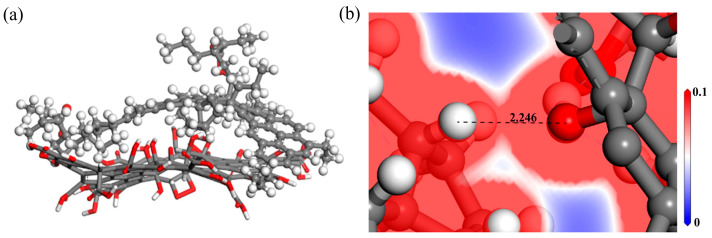
Mechanism of GO-modified asphalt: (**a**) adsorption; (**b**) hydrogen bonding interaction. Adapted with permission from Ref. [82]. Copyright Elsevier.

The modification mechanism of GO is depicted in Figure 14, with several viewpoints: (1) from a molecular structural perspective, the surface of GO contains a large number of oxygen-containing functional groups such as carboxyl (-COOH), hydroxyl (-OH), and epoxy groups, reducing the van der Waals forces between GO molecules and making it easily soluble in asphalt [83]; (2) oxygen molecules form hydrogen bonds with asphalt molecules, generating intermolecular van der Waals forces, and interact through aromatic stacking with aromatics and resins [82]. Additionally, GO exhibits electrostatic properties, enabling interaction with asphalt molecules through electrostatic forces; (3) as an asphalt modifier, GO increases surface roughness, effectively restricting aggregate movement and reducing deformation under various stress conditions; (4) the layered structure of GO increases the contact area with asphalt, allowing rapid adsorption of colloids and asphaltene, thereby enhancing asphalt viscosity. Regarding physicochemical reactions, some researchers believe that GO does not undergo chemical reactions during the modification process, but rather disperses into the asphalt matrix through physical blending, influencing the asphalt’s performance through its inherent properties [73]. However, other researchers argue that GO’s modification of asphalt involves a combined effect of physical and chemical reactions [84].

### 4.3. Nanoclay

**Figure 15:**
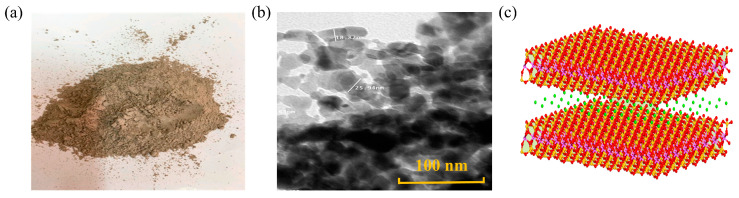
Shape and structure of NC: (**a**) macroscopic scale; (**b**) microscale; (**c**) molecular scale. Adapted with permission from Ref. [90]. Copyrights 2023 MDPI.

NC, belonging to layered silicates, includes montmorillonite (MMT), rectorite (REC), kaolinite, and organophilic bentonite, among which MMT is the most commonly used, as shown in Figure 15. The primary advantage of NC lies in their abundant availability in nature and low production costs. Qadir [85] evaluated the absolute viscosity of NC-modified asphalt and compared it with matrix asphalt. The observed viscosity increased from 242 cP for 0 wt.% NC content to 498.4 cP for 5 wt.% NC content. The increase in viscosity is attributed to the large surface area of NC, which enhances the adsorption of asphalt molecules on the NC surface. Additionally, NC forms a network within the asphalt, restricting the movement of asphalt molecules, making flow more difficult, and consequently resulting in higher viscosity. Patra [86] investigated the viscosity of NC-modified asphalt and observed a significant increase in viscosity as the NC dosage increased from 3 wt.% to 4.5 wt.%. Beyond this value, there was no significant increase. The increase in viscosity is attributed to the aggregation of NC within the asphalt, forming a network structure that impedes the movement of asphalt molecular chains and enhances shear resistance [87,88]. However, regarding the dosage of NC, Yousif has drawn different conclusions [89]. They believe that modified asphalt with an NC content of 7 wt.% exhibits higher viscosity values. The reason is that the peeling structure hinders the movement of modified asphalt molecular chains, and the dispersion of NC increases the shear resistance of asphalt molecules.

## 5. High Polymers

### 5.1. Styrene-Butadiene-Styrene

**Figure 16:**
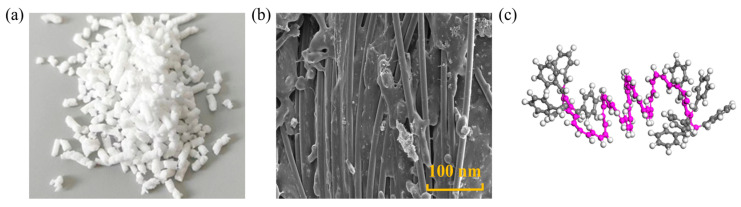
Shape and structure of SBS: (**a**) macroscopic scale; (**b**) microscale; (**c**) molecular scale. Adapted with permission from Refs. [94,95,96]. Copyrights 2020 Elsevier, 2023 Walter de Gruyter, and 2021 John Wiley and Sons Inc.

SBS is the most widely used asphalt modifier, generally appearing as white granules, and is a thermoplastic elastomer [91,92]. The shape and structure of SBS are depicted in Figure 16. Dong [91] observed that the addition of SBS significantly reduced the diffusion of asphalt molecules, especially saturate and aromatics, by 78.2 wt.% and 76.7 wt.%, respectively. For the microstructure, Liu [11] believed that SBS selectively absorbed saturated and aromatic fractions, disrupting the original colloidal structure of asphalt and forming new structures. When the SBS content was less than 4 wt.%, it dispersed well in asphalt and came into close contact with asphaltene, with enough small asphalt molecules around it. However, when the SBS content reached and exceeded 7 wt.%, it disrupted the original colloidal structure of the asphalt. Due to intermolecular forces, SBS molecules separated from asphalt micelles and aggregated. Xu [93] indicated that regardless of the degree of asphalt aging, the viscosity of SBS-modified asphalt was more significant than that of base asphalt. The viscosity calculated through MD was 5~9 cP, which fell within the previous simulation results. The simulation and experimental results were consistent and could be used for aging analysis. These findings emphasize the multifaceted effects of SBS on asphalt performance, including its impact on molecular diffusion, microstructural changes, and viscosity variations.

### 5.2. Styrene-Butadiene Rubber

**Figure 17:**
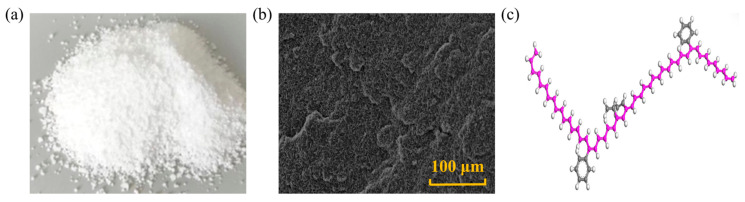
Shape and structure of SBR: (**a**) macroscopic scale; (**b**) microscale; (**c**) molecular scale. Adapted with permission from Refs. [95,101,102]. Copyrights 2023 Walter de Gruyter and 2024 MDPI.

**Figure 18:**
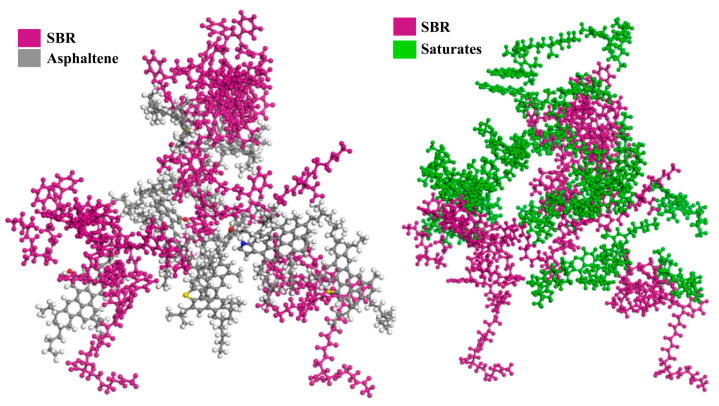
Cross-linked network between SBR and asphalt molecules. Adapted with permission from Ref. [97]. Copyrights 2024 Elsevier.

SBR possesses excellent wear resistance, heat resistance, and aging properties, and is widely used in the production of tires, cables, medical equipment, and various rubber products. Currently, SBR is extensively employed as an asphalt modification material in road construction, with its shape and structure depicted in Figure 17. To evaluate the rheological properties of SBR-modified asphalt, Xie [97] studied the effects of modified asphalt on different SBR contents through MD simulations and free volume theory. The results indicated that with the increase in SBR, the proportion of free volume in the modified asphalt decreased, leading to increased viscosity. When the SBR content reached 30 wt.%, some SBR molecules would aggregate and cause phase separation, affecting the colloidal stability. In practical engineering applications, it is recommended to limit the SBR content to around 20 wt.%. Several explanations for modification of SBR are as follows: (1) Free volume provides space for molecular movement [98], and under the influence of SBR chains, molecular thermal movement decreases, compacting the asphalt structure; (2) SBR is prone to swelling reactions, absorbing some light components in the asphalt, such as aromatics, promoting the formation of a cross-linked network [99,100], as shown in Figure 18. This action hinders the flow of surrounding asphalt macromolecules at high temperatures, enhancing the asphalt’s resistance to high-temperature deformation.

## 6. Composites

### 6.1. Nanomaterials

**Figure 19:**
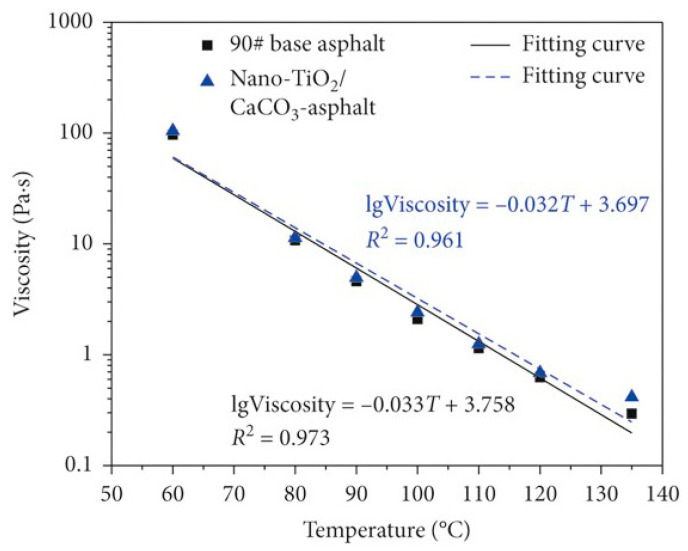
Viscosity temperature curves of matrix asphalt and NT/NCa-modified asphalt. Adapted with permission from Ref. [103]. Copyrights 2021 Hindawi.

Zhang [103] conducted viscosity tests on NT/NCa-modified asphalt, and the results are depicted in Figure 19. It was observed that the viscosity of modified asphalt at 135 °C did not exceed 3 Pa∙s, ensuring its processability and construction performance. The addition of NT/NCa was found to be advantageous in enhancing the viscosity of the asphalt, as the nanoparticles absorb light components of the asphalt, increasing the ratio of resin and asphaltene, as well as adhesion. Furthermore, a reasonable content of NT/NCa was recommended to be 5 wt.% of the weight of the base asphalt. In Shafabakhsh’s study [20], NT/NS were introduced into asphalt mixtures, which caused an average 250 wt.% increase in the viscosity of the modified asphalt at temperatures ranging from 120 °C to 160 °C. This increase was attributed to the high surface area of NT/NS, which was found to increase the roughness and reactivity of the modified asphalt. Composite-modified asphalt was prepared by Fu [104] using varying contents of NT/NZ and basalt fibers. Experimental results indicated that with the addition of the modifier, the ZSV of the modified asphalt increased at the same temperature, which was beneficial for high-temperature stability and resistance to deformation. Additionally, FTIR results indicated that there were no chemical reactions between basalt fibers, NT/NZ, and asphalt, suggesting a primarily physical modification mechanism. The optimal content of NT/NZ in the asphalt was found to be 4 wt.%, while the basalt fiber content was 6 wt.%. The uniform distribution of nanoparticles in the asphalt, together with the network structure formed by basalt fibers, was noted to impede the diffusion of internal microcracks. Duan [105] investigated the influence of different ZnO/layered silicate composite materials on the physical properties of asphalt, conducting penetration, softening point, viscosity, and ductility tests. It was found that after introducing the ZnO/layered silicate composite materials, the penetration of the modified asphalt decreased, while the softening point and viscosity increased compared to the base asphalt. This was attributed to the formation of an embedded phase structure. Specifically, when the composite material was added to the asphalt, the more flowable light components in the asphalt entered the voids of the composite material and increased the hardness of the asphalt.

The above studies collectively highlight the diverse effects of different nanoparticles on asphalt properties, emphasizing their role in enhancing viscosity, high-temperature stability, and resistance to deformation, showcasing a diverse range of nanoparticle influences on asphalt behavior.

### 6.2. Nanomaterials/SBS

#### 6.2.1. 0D Nanomaterials/SBS

**Figure 20:**
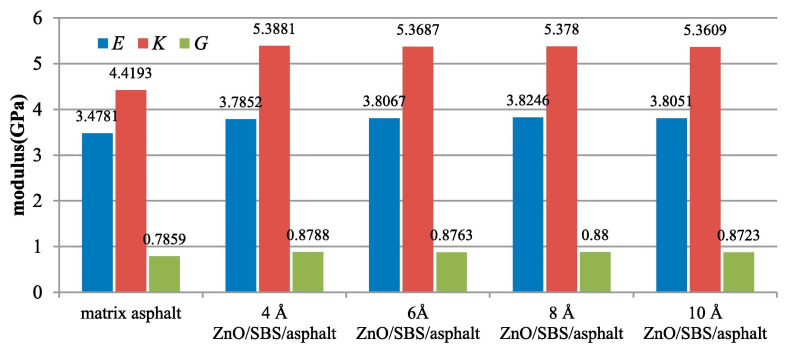
Physical moduli of asphalt and NZ/SBS/asphalt. Adapted with permission from Ref. [94]. Copyrights 2020 Elsevier.

Through MD simulation, Su [94] studied the compatibility of NZ with SBS and asphalt, as well as the influence of SBS on the diffusion coefficient of NZ in asphalt. The results indicated that NZ reduced the solubility parameter of asphalt and enhanced the compatibility between SBS and asphalt. As shown in Figure 20, NZ/SBS increased the bulk modulus (*K*), shear modulus (*G*), and elastic modulus (*E*) of asphalt, leading to a denser asphalt structure, increased viscosity, and thereby improved the high-temperature performance of asphalt. During the modification process, SBS dispersed in the form of fine particles within the asphalt. Upon the addition of NZ, the vacant orbitals in Zn atoms could accept electrons from the benzene rings in SBS and the aromatic rings in the base asphalt. This interaction facilitated cross-linking between SBS and the base asphalt, promoting reactions between the functional groups of internal polymers and enhancing the stability of the modified asphalt. This also suggests that the ZnO/SBS composite-modified asphalt underwent physical and chemical modification. Physical modification primarily occurred between asphalt and SBS, while chemical modification mainly occurred between asphalt and ZnO [106,107]. Huang [108] found that the permeability of NS/SBS-modified asphalt decreased and the viscosity increased with an increase in the NS content. This was attributed to the strong adsorption capacity of NS particles, which enhanced the network structure of SBS-modified asphalt and increased the flow resistance of asphalt molecules. Additionally, FTIR analysis showed that after adding NS to SBS-modified asphalt, the peaks of NS/SBS-modified asphalt were almost identical to those of SBS-modified asphalt, indicating that NS primarily bridged through physical adsorption in SBS-modified asphalt without significant chemical reactions. Sun [109] studied the performance of NA/SBS-modified asphalt at 135 °C, demonstrating that NA could improve the high-temperature performance and bonding properties of SBS asphalt while maintaining its low-temperature performance. Chromatograms and analysis of the weight-average molecular weight showed an increase in the molecular weight of polymer groups and asphalt macromolecular groups in NA-modified SBS asphalt. AFM analysis revealed an increase in the number, volume, and roughness of honeycomb structures in SBS asphalt after NA modification. No new absorption peaks were observed in the FTIR analysis, indicating that the modification of NA/SBS asphalt occurred through physical blending. Sun [110] analyzed the high-temperature performance of NCa/SBS-modified asphalt, indicating that the optimal performance was achieved when the amount of NCa was 4 wt.%. Compared to SBS-modified asphalt, the viscosity of NCa/SBS-modified asphalt increased, and the dynamic stability improved by 16.4 wt.%, further enhancing the high-temperature rutting resistance of the mixture.

These studies highlight the potential of different nanoparticles/SBS-modified asphalt. Although the types of nanoparticles focused on are different, they consistently indicate the improvement of the physical and high-temperature properties of modified asphalt. Meanwhile, nanoparticles help promote the interaction between SBS and base asphalt, thereby achieving physical and chemical modifications in modified asphalt.

#### 6.2.2. 1D Nanomaterials/SBS

**Figure 21:**
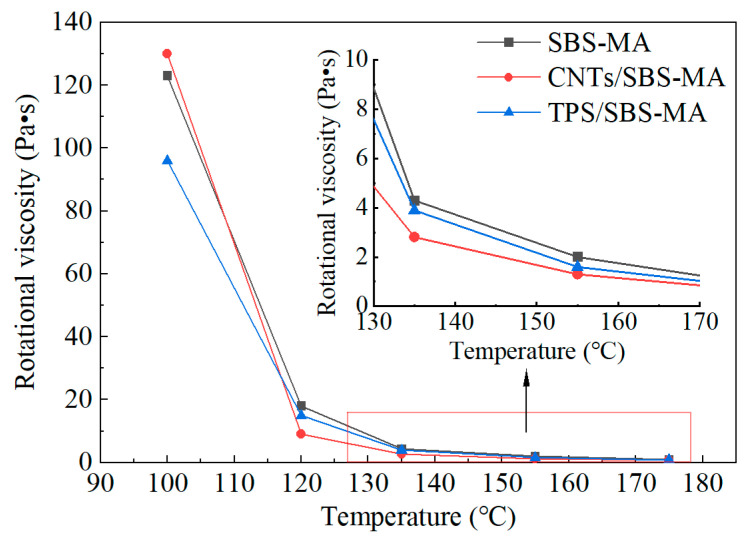
Viscosity–temperature relationship curves of three types of asphalt. Adapted with permission from Ref. [111]. Copyrights 2022 MDPI.

Chen [111] prepared a composite-modified asphalt using a surface-modified CNT and SBS modifier. The influence of CNT on the properties of SBS-modified asphalt was investigated through rheological experiments. As depicted in Figure 21, the experimental results indicated that at temperatures ranging from 100 °C to 120 °C, the viscosity of CNT/SBS-modified asphalt was higher than that of SBS-modified asphalt. However, at a test temperature of 135 °C, the viscosity of CNT/SBS-modified asphalt was lower than that of SBS-modified asphalt, meeting the processable specification of less than 3 Pa·s. This was attributed to the high polarity of hydroxylated II-CNT, which reduced the viscosity of the modified asphalt. Therefore, at temperatures below 100 °C, CNT/SBS-modified asphalt exhibited high viscosity and excellent resistance to high-temperature rutting. At construction temperatures exceeding 120 °C, CNT/SBS-modified asphalt demonstrated low viscosity, good flowability, and processability. Goli [112] investigated the impact of CNT on the compatibility of SBS-modified asphalt. The results indicated that the addition of CNT increased the complex modulus of SBS-modified asphalt and reduced the phase angle. Compared to the control asphalt, the difference between the top and bottom rheological parameters of CNT/SBS/base asphalt decreased, suggesting that the addition of CNT enhanced the viscosity and storage stability of SBS-modified asphalt. The interface region between SBS and the asphalt formed aromatic fractions, and the addition of CNT strengthened the molecular interaction in the interface region, enhancing the performance of the asphalt in a fibrous network manner. Additionally, CNT formed π–π conjugates with aromatic components and intertwined with the alkane chains in the asphalt, SBS, and CNT, further reinforcing the interfacial interaction, thereby enhancing stability.

#### 6.2.3. 2D Nanomaterials/SBS

**Figure 22:**
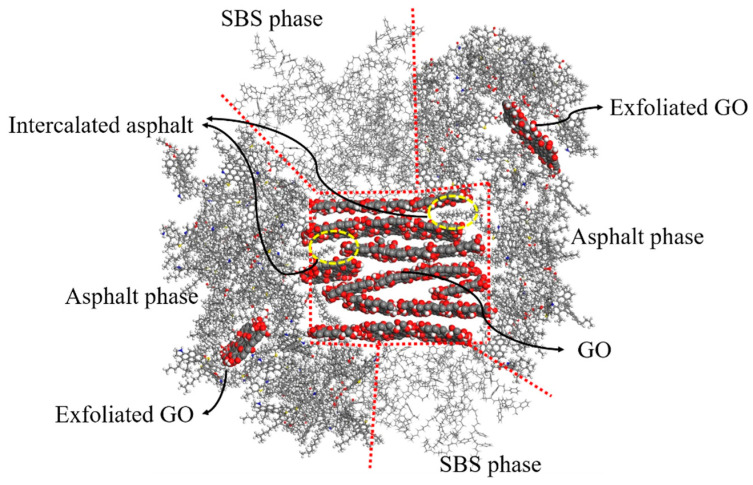
Interface microstructure of GO/SBS-modified asphalt. Adapted with permission from Ref. [114]. Copyrights 2023 Springer Nature.

Mo [113] studied GO/SBS-modified asphalt using high-speed shearing, and the influence of GO on the physical properties of SBS-modified asphalt was investigated. According to the results, with increasing GO content, the penetration value of the GO/SBS-modified asphalt initially decreased and then increased, indicating that an appropriate amount of GO could reduce the penetration value of SBS-modified asphalt, increase viscosity, and enhance the asphalt’s resistance to deformation. The optimal modification effect was observed when the GO content was 0.75 wt.%. Zeng [114] combined experimental and MD simulation methods to study the synergistic modification effect and interface mechanism of the modifier with asphalt. The results indicated that the modification effect of GO/SBS blended into the asphalt was significantly superior to individually adding GO or SBS. GO could simultaneously combine with SBS and asphalt, enhancing the binding strength and promoting the dispersion of SBS in the asphalt, which demonstrates a synergistic modification effect to improve macroscopic rheological and storage stability properties. As shown in Figure 22, the intercalation and exfoliation structure formed between GO and asphalt significantly hindered the movement of asphalt molecular chains [114].

Galooyak [115,116] employed a melt blending method to prepare organo-nanoclay (OMMT)/SBS-modified asphalt and compared the physical and rheological properties of SBS-modified asphalt before and after the addition of OMMT. The results indicated that the presence of OMMT significantly enhanced the storage stability of SBS-modified asphalt. Characterization of the structure of the OMMT/SBS/asphalt blend was conducted using X-ray diffraction (XRD), revealing that the appropriate dispersion of OMMT resulted in a uniform blend. The viscosity of the modified asphalt at 60 °C and 135 °C was measured using a Brookfield viscometer, and it was found that the viscosity increased with the addition of OMMT and SBS. The phenomenon was attributed to the restriction of asphalt molecular chain movement at high temperatures. The modification mechanism of OMMT/SBS-modified asphalt primarily involves physical interactions [108]. On one hand, the interaction between asphalt and SBS leads to the exfoliation of OMMT, resulting in the formation of numerous cross-linking points due to the presence of intermolecular forces. On the other hand, SBS results in swelling, forming a spatial network cross-linking structure.

The aforementioned studies emphasize that when modifiers such as GO and OMMT are added to asphalt, they consistently enhance viscosity, indicating improved resistance to deformation and increased storage stability. Furthermore, apart from the synergistic modification effect, different materials exhibit strengthened binding strength. Structurally, the presence of modifiers hinders the movement of asphalt molecular chains and results in the formation of uniform blends. These findings collectively underscore the multifaceted benefits of incorporating modifiers in SBS-modified asphalt, leading to improved rheological properties.

### 6.3. Nanomaterials/SBR

**Figure 23:**
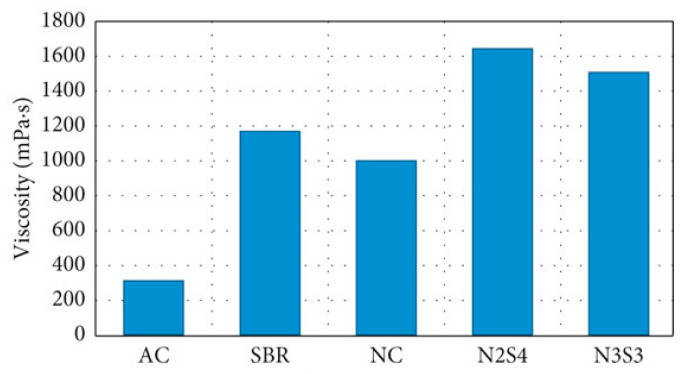
Viscosity of modified asphalt with different modifiers. Adapted with permission from Ref. [119]. Copyrights 2018 Hindawi.

Li [117] prepared NCa/NZ/SBR-modified asphalt and studied the influence of the modifier on the pavement performance. The results indicated that the optimal combination for the modified asphalt was 4 wt.% NCa + 5 wt.% NZ + 4 wt.% SBR. At 82 °C, the asphalt shear modulus increased by 24.1 wt.%, and the viscosity increased by 14.6 wt.% to 23.1 wt.%. Through FTIR testing, chemical reactions primarily occurred between the asphalt and the nanomaterials, while physical changes took place within the SBR. Xue [118] further modified the asphalt containing 4 wt.% SBR using CNT. According to the experimental results, CNT easily condensed and precipitated with SBR at high temperatures, improving the rheological properties of the bottom of the asphalt. When the CNT content was low, the SBR-modified asphalt could not form a dense structure. With an increase in CNT content, 1.0 wt.% and 1.5 wt.% of CNT made the SBR-modified asphalt more compact, and the higher cross-linking density played a role in inhibiting molecular diffusion to increase the viscosity. FTIR testing indicated that the reaction in CNT/SBR/asphalt composite belonged to physical blending. As shown in Figure 23, based on the research results of Ameri [119], the viscosity of NC/SBR-modified asphalt in the base asphalt was higher than that of singly modified SBR or NC asphalt, and the 2 wt.% NC + 4 wt.% SBR (N2S4)-modified asphalt exhibited higher viscosity performance.

## 7. Challenges and Outlook

### 7.1. Compatibility and Dispersion

The compatibility and dispersion of nanomaterials (dispersed phase) within the asphalt matrix (continuous phase) are critical factors for realizing the superior performance of nanomodified asphalt. The large surface area and strong hydrophilicity of nano modifiers make them prone to aggregation and separation from asphalt, diminishing or even negating their positive effects on asphalt performance [15,120]. The uneven dispersion of nanomaterials can lead to local enrichment or segregation, causing structural distortion and uneven force fields in the modified asphalt. To address these issues, surface modifiers have been an effective solution [15]. Common surface modifiers include KH-570, APTES, and KH-560, used for surface modification of NS, NT, and NZ, respectively [120]. Another approach to tackle nanomaterial aggregation and improper dispersion is by selecting suitable blending methods. The melt blending method is simple and efficient, more easily applicable on an industrial scale, but the uniformity of the modified asphalt system is not as good as that of the solution method. Utilizing the “bridging” effect of solvents, the solution method can achieve the uniform dispersion of nanomaterials in asphalt. However, this process is complex and adds additional production costs. Therefore, the melt blending method is still commonly used for the production of nano-modified asphalt, and high mixing temperatures can promote the diffusion of nanomaterials in asphalt by reducing asphalt viscosity.

### 7.2. Cost

The high cost of nanomaterials makes their use as asphalt modifiers uneconomical at high dosages, primarily due to construction costs. Additional expenses depend on the type, quantity, size, or purity of the nanomaterials [121]. Given the use of expensive equipment and technology, it is understandable that their prices are higher than traditional paving materials. However, over time, the cost of nanomaterials is expected to decrease, and the use of substitutes and improvements in manufacturing technology will continue this trend. From an economic perspective, modifiers with lower optimal dosages are more competitive. Table 2 summarizes the data from various studies, showing the types, contents, sizes, and costs of several nanomaterials. Analysis shows that compared to other materials, NS, NC, and SBS have relatively lower costs, so these materials may have more prospects as asphalt modifiers. Meanwhile, composite-modified asphalt mainly composed of these materials also has prospects. In addition to the direct costs of nanomaterials, there are additional indirect costs associated with nanomodification, including equipment expenses and the hiring of experienced personnel [122].

### 7.3. Environment

In the process of synthesizing nanomaterials, various organic solvents (such as benzene and ethers), surfactants, as well as metal and heavy metal ions (such as selenium and zinc), are extensively utilized, leading to the presence of impurities within them [127]. The construction and laying of asphalt pavements are conducted in open-air environments, where some nanoparticles may disperse outdoors, resulting in the generation of toxic air and potentially causing a variety of health issues [121].

### 7.4. Further Research

In response to current challenges and difficulties, the development and utilization of novel nanomaterials and composite nanomaterials to enhance asphalt performance have become a crucial area of focus. While most research is concentrated on the fundamental properties of nanomaterial-modified asphalt, the development of functional nanomodified asphalt is an aspect worthy of attention. Simultaneously, addressing the poor dispersion of nanomaterials in asphalt can significantly improve the service performance of asphalt mixtures. Introducing new dispersion and characterization technologies, such as ultrasonic dispersion and X-ray computed tomography (XCT), offers the possibility of further uniformly dispersing nanomaterials into asphalt. Another research focus is the life cycle cost analysis of modified asphalt, encompassing operational costs, maintenance, repair, energy consumption, and other factors.

## 8. Conclusions

In summary, this review elucidates the modifying effects of various nanomaterials on asphalt viscosity, summarizes challenges such as poor compatibility and dispersibility, high production costs, and environmental and health issues, and emphasizes the necessity of conducting economic and ecological assessments of nanomodified asphalt before large-scale implementation, providing technical and practical significance for sustainable road asphalt material design. The main conclusions are as follows:

1. Nanomaterials, as modifiers, tend to increase the softening point and viscosity of asphalt, thereby improving performance in high-temperature rutting resistance and low-temperature cracking resistance. Modification mechanisms include both physical and chemical methods, with physical modification being predominant.
2. The modification process of 0D nanomaterials on asphalt involves their high activity and surface energy. They firmly attach to the asphalt surface, enhancing interactions with asphalt molecules and reducing their mobility. Additionally, they chemically combine with asphalt molecules to form hydrogen bonds and create a network structure.
3. The modification process of CNT on asphalt involves interactions between p electrons and non-polar asphalt molecules, forming bonds between the tube walls. This enhances adhesion and stability, providing resistance to cyclic loading. The modification mechanism of CNF on asphalt varies: high porosity and absorption reinforce asphalt, high specific surface area induces expansion, and bundle-shaped nanofibers enhance and toughen asphalt, delaying crack propagation within asphalt mixtures.
4. Graphene has strong adsorption capabilities, enhancing rheological properties through interactions with polycyclic aromatic hydrocarbons. It increases asphalt viscosity and compactness, transitioning asphalt’s structure to a gel form. GO, characterized by oxygen-containing groups, enhances solubility in asphalt, reduces van der Waals forces, and engages in chemical interactions, forming hydrogen bonds and aromatic stacking. Additionally, it increases surface roughness, restricts aggregate movement, and enhances asphalt viscosity. NC impedes asphalt molecular chain movement, forms a network structure, and significantly improves asphalt binder properties.
5. The polymer’s molecular chains reduce the thermal movement of asphalt molecules, compacting the structure and promoting the formation of a cross-linked network by absorbing light components. This enhances the asphalt’s resistance to high-temperature deformation. Composite-modified materials combine the strengths of each material, offering greater potential to enhance asphalt performance.
6. Nanomaterial-modified asphalt encounters challenges including poor compatibility and dispersibility, high production costs, and environmental and health concerns. Conducting economic and ecological evaluations before large-scale application in practical engineering is crucial. Analysis reveals that, in comparison to other materials, NS, NC, and SBS have relatively lower costs and thus hold more promise. Moreover, integrating low-cost materials with others can further reduce economic expenses.

The review’s ideas originate from the author’s original research and experience, free from plagiarism. The main limitation is the scarcity of literature dedicated to the viscosity of nanomaterial-modified asphalt. Future studies should focus on delving deeper into molecular interactions between nanomaterials and asphalt binders to gain a more nuanced understanding of underlying mechanisms. Additionally, efforts should be directed towards standardizing testing protocols for assessing the effectiveness of different nanomaterials in asphalt modification, enabling comparative evaluations across various studies.

## Figures and Tables

**Table 2:** Costs of different types of nanomaterials.

Nanomaterials	Content (wt.%)	Size (nm)	Surface Area (m^2^/g)	Cost ($/kg)	Time (AD)	Ref.
NZ	3	-	-	150	2023	[17]
NS	-	5~20	≥690	1092.4~1638.6	2019	[123]
	-	15~70	130~600	8.74~163.86		
	1	-	-	1.57	2018	[17]
	3	-	-	5.34	2021	[124]
	5	-	-	15.99	2017	[14]
NFe (purity:98 wt.%)	-	50	-	109.24~327.72	2019	[123]
NC	-	-	-	109.24~273.1	2019	[123]
	2	-	-	4.96	2015	[14]
	10	-	-	18	2023	[17]
CNT	1.5	-	-	50~110	2021	[60]
GO	1.5	-	-	533~793	2020	[28]
	-	-	-	481~1350	2024	[125]
SBS	-	-	-	2.26	2022	[126]

## Data Availability

The data that support the findings of this study are available from the corresponding authors upon reasonable request.
